# Neonatal Maternal Deprivation Followed by Adult Stress Enhances Adrenergic Signaling to Advance Visceral Hypersensitivity

**DOI:** 10.1007/s12264-018-0318-3

**Published:** 2018-12-17

**Authors:** Wan-Jie Du, Shufen Hu, Xin Li, Ping-An Zhang, Xinghong Jiang, Shan-Ping Yu, Guang-Yin Xu

**Affiliations:** 1 Laboratory for Translational Pain Medicine, Institute of Neuroscience, Soochow University, Suzhou, 215123 China; 2 Center for Translational Medicine, The Zhangjiagang Affiliated Hospital of Soochow University, Zhangjiagang, 215600 China; 3 Department of Anesthesiology, Emory University School of Medicine, Atlanta, GA 30307 USA

**Keywords:** Irritable bowel syndrome, Dorsal root ganglion, Norepinephrine, Visceral pain, Stress

## Abstract

The pathophysiology of visceral pain in patients with irritable bowel syndrome remains largely unknown. Our previous study showed that neonatal maternal deprivation (NMD) does not induce visceral hypersensitivity at the age of 6 weeks in rats. The aim of this study was to determine whether NMD followed by adult stress at the age of 6 weeks induces visceral pain in rats and to investigate the roles of adrenergic signaling in visceral pain. Here we showed that NMD rats exhibited visceral hypersensitivity 6 h and 24 h after the termination of adult multiple stressors (AMSs). The plasma level of norepinephrine was significantly increased in NMD rats after AMSs. Whole-cell patch-clamp recording showed that the excitability of dorsal root ganglion (DRG) neurons from NMD rats with AMSs was remarkably increased. The expression of β_2_ adrenergic receptors at the protein and mRNA levels was markedly higher in NMD rats with AMSs than in rats with NMD alone. Inhibition of β_2_ adrenergic receptors with propranolol or butoxamine enhanced the colorectal distention threshold and application of butoxamine also reversed the enhanced hypersensitivity of DRG neurons. Overall, our data demonstrate that AMS induces visceral hypersensitivity in NMD rats, in part due to enhanced NE-β_2_ adrenergic signaling in DRGs.

## Introduction

Irritable bowel syndrome (IBS) is a common gastrointestinal disease characterized by disorders of intestinal motility and accompanied by chronic abdominal pain [1–3]. The treatment of chronic abdominal pain is difficult [4]. Research on this disease is progressing slowly due to the lack of suitable animal models. Although the currently available research provides insights into the processing and regulation of chronic pain [5, 6], the precise pathophysiology of IBS has not been fully elucidated and effective strategies for treating the primary symptoms are not available [1, 7, 8]. Our previous studies have shown that neonatal maternal deprivation (NMD) can induce chronic visceral pain in adult rats at the age of 7 weeks but not at 6 weeks [9, 10]. It seems that the age of 7 weeks is an important time point for the development of visceral hypersensitivity in rats with NMD. However, it is unknown whether 6-week-old NMD rats are more sensitive to environmental stimuli than age-matched controls.

A growing body of evidence has demonstrated that severe adverse environmental factors such as stress might be a stimulus to generate visceral hypersensitivity at the adult age in humans [11, 12] and animals [13–15]. For example, repetitive water-avoidance stressors for 10 days induce visceral hypersensitivity in rats and mice [16]. Heterotypic intermittent stress for 9 consecutive days induces visceral hypersensitivity immediately after termination of the last stressors [14, 17]. However, single or mild stressors do not induce visceral hypersensitivity in healthy adult animals. In the present study, we designed a stress protocol to determine whether one stressor or mild stressors could induce visceral pain in NMD rats at the age of 6 weeks.

Adrenergic signaling plays many important roles in the nervous system to regulate stress responses [18–20]. Adrenergic receptors (ARs) are classically divided into two main groups: α- and β-adrenoceptors. The α-adrenoceptors include α_1A_, α_1B_, α_1D_, α_2A_, α_2B_, and α_2C_ subtypes, and β-adrenoceptors into β_1_, β_2_, and β_3_ subtypes [21]. They have been reported to be expressed in primary sensory neurons with their cell bodies located in the dorsal root ganglia (DRGs) [22, 23]. Previous studies have suggested that the β_1_ and β_2_ subtypes are involved in the adrenergic activation [24] that may play a role in colonic transit. The β_2_ ARs are reported to produce a hyperalgesic state in rats [22, 25, 26]. The β_3_ ARs, mainly expressed in brown and white adipose tissue, regulate energy metabolism and thermogenesis [27]. Besides, other studies have shown that the adrenergic system plays a role in the visceral pain caused by chronic stress [13]. Recently, we have reported that adrenergic β_2_ receptors mediate visceral pain evoked by heterotypic intermittent stress in rats [14]. However, whether adrenergic signaling in primary sensory neurons participates in visceral pain of NMD rats at the age of 6 weeks after additional adult stress is unknown.

Thus, we designed this study to test the hypothesis that adrenergic activation plays a crucial role in the switch of NMD rats at the age of 6 weeks from no pain status to pain hypersensitivity induced by stress in adulthood.

## Materials and Methods

### Animals

We used male Sprague-Dawley rats in the present experiments. The experimental protocol was approved by the Institutional Animal Care and Use Committee of Soochow University. Animal care and handling were strictly in accordance with the regulations and guidelines of the International Association for the Study of Pain.

### Adult Stress Protocol to Induce Visceral Pain

**Fig. 1:**
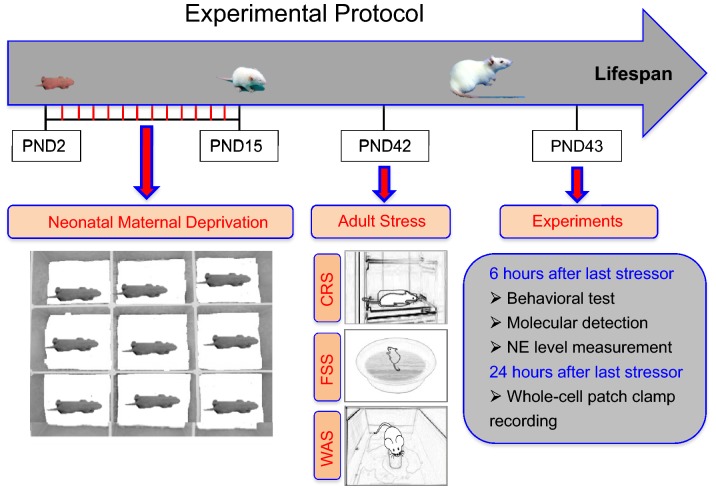
Cartoon showing the experimental protocol for inducing visceral hypersensitivity. This protocol had two parts: neonatal maternal deprivation (NMD) and adult stress. NMD was imposed from postnatal day (PND) 2 to 15. The adult stress was of two kinds: adult single stressor (ASS): cold-restraint stress (CRS) for 45 min, and adult multiple stressors (AMSs): CRS for 45 min, forced-swimming stress (FSS) for 20 min, and water-avoidance stress (WAS) for 60 min. Behavioral testing was performed 6 h and 24 h after the termination of the last stressor. Molecular detection and norepinephrine (NE) measurements were performed 6 h after the termination of the last stressor. Whole-cell patch-clamp recordings were made 24 h after termination of the last stressor.

The stress protocol to induce chronic visceral hyperalgesia is shown in Fig. 1. This protocol had two parts, NMD and stress in adulthood. The adult stress was divided into an adult single stressor (ASS), cold-restraint stress (CRS), for 45 min, and adult multiple stressors (AMSs) consisting of CRS for 45 min, forced-swimming stress (FSS) for 20 min, and water-avoidance stressor (WAS) for 60 min. The interval between each stressor was 60 min. NMD was imposed as described previously [9, 10, 28]. In brief, pups were separated from the maternity cages and placed in different cages with an electric blanket to maintain body temperature at ~32 °C for 3 h every day from postnatal days 2 to 15. After the 3 h of separation, pups were returned to the dam cages. Littermates in the control group were not handled and were kept in the maternity cages with their dam. Both groups were exposed to an ASS or AMSs on postnatal day 42 (Fig. 1). All experiments were performed 6 h or 24 h after termination of last stressor unless indicated otherwise. Multiple batches of rats at the age of 6 weeks were used in the present study. The numbers of animals in each group were as follows: control group, 15; NMD group, 17; CON + ASS group, 13; NMD + ASS group, 16; CON + AMS group, 38; and NMD + AMS group, 68.

### Measurement of Behavioral Responses to Graded Colorectal Distention (CRD)

Chronic visceral hyperalgesia was assessed by grading the behavioral response of rats to CRD at the age of 6 weeks based on previous publications [9, 10, 20]. All behavioral tests were performed in a blinded manner.

### Drug Administration

In the behavioral experiments, 5 mg/kg butoxamine (BUTO, a β_2_ antagonist; Sigma, St. Louis, MO), 3 mg/kg propranolol (PROP, a norepinephrine β receptor antagonist; Sigma) or 3 mg/kg phentolamine (PHEN, an α receptor antagonist; Sigma) dissolved in 0.9% normal saline (NS) was intraperitoneally injected into AMS rats once for behavioral experiments and once daily for 7 consecutive days for patch-clamp recordings and western blotting. The drug concentrations used were based on our previous study and reports from other groups [14, 20].

### Measurement of Norepinephrine (NE) in Blood Plasma

Blood samples were collected from the trunk into centrifuge tubes containing 0.45% citric acid and 2.5% sodium citrate at euthanasia by decapitation. After refrigerated centrifugation, the supernatant was quickly aliquoted and stored at − 80 °C for experiments. NE levels in the plasma were determined using an enzyme immunoassay kit from Abnova (Norepinephrine ELISA Kit), as previously described [20].

### Western Blotting

DRGs at T_13_–L_2_ from AMS-treated control or NMD rats were collected to measure the protein levels of β_1_, β_2_, and β_3_ receptors. DRGs at T_13_–L_2_ from control, NMD, and ASS-treated control or NMD rats were collected to measure the protein levels of β_2_ receptors. The antibodies used were anti-β_1_ (1:500, Santa Cruz Biotechnology, Inc.), anti-β_2_ (1:500, Santa Cruz Biotechnology, Inc.), anti-β_3_ (1:500, Santa Cruz Biotechnology, Inc.), and anti-GAPDH (1:2000, Hangzhou Goodhere Biotechnology). Band density was measured using ImageJ software. The β_1_, β_2_, and β_3_ expression were normalized to GAPDH as described previously [17, 20, 29, 30].

### Real-Time qPCR

Total RNAs were extracted from DRGs (T_13_–L_2_) from control, AMS, and NMD rats with TRIzol (Ambion, Shanghai, China). cDNA was synthesized from total RNA using a reverse transcription kit (Transgen Biotech, Beijing, China) according to the supplier’s instructions. The primers are as follows: *β*_*2*_ forward 5′-GGTTGGGCTATGTCAACTCTG-3′, reverse 5′-GTCTGTCCTACCGTTGCTGTT-3′; *gapdh* (internal control) forward 5′-TGGAGTCTACTGGCGTCTT-3′, reverse 5′-TGTCATATTTCTCGTGGTTCA-3′. Control reactions were carried out without cDNA templates.

### Dissociation of DRG Neurons and Whole-Cell Patch-Clamp Recordings

Rats from AMS-treated NMD or control (~6 weeks) were sacrificed by decapitation. The detailed procedures for the acute isolation of DRG neurons and patch clamp recordings were as previously reported [14, 20, 31]. The dissecting solution contained (in mmol/L): 130 NaCl, 5 KCl, 2 KH_2_PO_4_, 1.5 CaCl_2_, 6 MgSO_4_, 10 glucose, and 10 HEPES, pH 7.2 with osmolarity 305 mOsm. For the patch-clamp recordings, normal external solution contained (in mmol/L): 130 NaCl, 5 KCl, 2 KH_2_PO_4_, 2.5 CaCl_2_, 1 MgCl_2_, 10 HEPES, 10 glucose, pH adjusted to 7.2 with NaOH, osmolarity 295–300 mOsm. The pipette solution contained (in mmol/L): 140 K-gluconate, 10 NaCl, 10 HEPES, 10 glucose, 5 EGTA, 1 CaCl_2_, pH 7.25 adjusted with KOH; osmolarity 292 mOsm.

### Data Analysis

All data are presented as mean ± SEM. Statistical testing was performed using OriginPro 8 (OriginLab, Northampton, MA). Normality was first checked for all data before analysis. Significance was determined using the two-sample *t* test, Mann-Whitney test, Mann-Whitney test following Friedman ANOVA, or Tukey’s *post-hoc* test following two-way repeated measures ANOVA. *P* < 0.05 was considered to be statistically significant.

## Results

### AMS Induces Visceral Hypersensitivity in NMD Rats

**Fig. 2:**
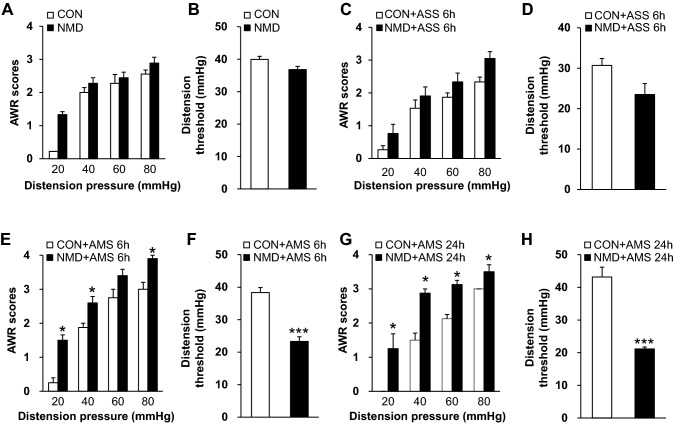
Multiple stressors induced chronic visceral hyperalgesia in NMD rats at 6 weeks. **A**, **B** NMD did not induce changes of distention threshold and AWR scores in response to colorectal distention (CRD) in 6 weeks, compared with controls. **C**, **D** NMD + ASS did not induce changes of distention threshold and AWR scores in response to CRD 6 h after ASS exposure, compared with CON + ASS rats. **E**, **F** NMD + AMS significantly reduced the distention threshold and increased AWR scores 6 h after AMS exposure when compared with CON + AMS. **G**, **H** NMD + AMS significantly reduced the distention threshold and increased AWR scores 24 h after AMS exposure compared with CON + AMS. AWR scores, *P < 0.05, Mann-Whitney test following Friedman ANOVA; DT, ***P < 0.001, two-sample *t* test.

Chronic visceral hyperalgesia was assessed as the abdominal withdrawal reflex (AWR) score and distension threshold (DT) in response to CRD at the age of 6 weeks. The NMD rats did not exhibit chronic visceral hyperalgesia at 6 weeks (Fig. 2A, B), consistent with previous studies [8, 29, 30]. Besides, ASS also did not induce chronic visceral hyperalgesia 6 h after CRS exposure in NMD rats at 6 weeks (Fig. 2C, D). However, the AWR scores increased significantly and DT decreased markedly after AMS exposure (Fig. 2E–H). The AWR scores were 1.50 ± 0.16, 2.60 ± 0.19, 3.40 ± 0.19, and 3.90 ± 0.10 in the NMD + AMS rats (*n* = 5 rats) and 0.25 ± 0.14, 1.88 ± 0.13, 2.75 ± 0.25, and 3.00 ± 0.20 in the CON + AMS rats (*n* = 5 rats) at 20 mmHg, 40 mmHg, 60 mmHg, and 80 mmHg distention pressure 6 h after AMS exposure, respectively. The AWR scores were 1.25 ± 0.43, 2.88 ± 0.13, 3.13 ± 0.13, 3.50 ± 0.20 in the NMD + AMS rats (*n* = 4 rats) and 0.00 ± 0.00, 1.50 ± 0.20, 2.13 ± 0.13, 3.00 ± 0.00 in the CON + AMS rats (*n* = 4 rats) at 20 mmHg, 40 mmHg, 60 mmHg and 80 mmHg distention pressure 24 h after AMS exposure, respectively. In addition, the DT was 38.33 ± 1.55 mmHg (*n* = 5 rats) and 23.33 ± 1.41 mmHg (*n* = 5 rats) 6 h after AMS exposure in the CON + AMS rats and NMD + AMS rats (Fig. 2F); and was 43.17 ± 3.02 mmHg (*n* = 4 rats) and 21.17 ± 0.57 mmHg (*n* = 4 rats) 24 h after AMS exposure in the CON + AMS rats and NMD + AMS rats (Fig. 2H). After statistical analysis, there was a remarkable difference in AWR scores and DT between CON + AMS and NMD + AMS rats (AWR scores, *P* < 0.05, Mann-Whitney test following Friedman ANOVA; DT, *P* < 0.001, two-sample *t* test). These data showed that the AWR scores were markedly greater in the NMD + AMS groups at 20 mmHg, 40 mmHg, 60 mmHg, and 80 mmHg distention pressures than those of age-matched CON + AMS rats 6 h and 24 h after AMS exposure. Meanwhile, the DT was greatly decreased in NMD + AMS rats compared with age-matched CON + AMS rats 6 h and 24 h after AMS exposure, indicating that AMS induced visceral pain in NMD rats at the age of 6 weeks.

### AMS Increases NE Concentration in Blood Plasma of NMD Rats

**Fig. 3:**
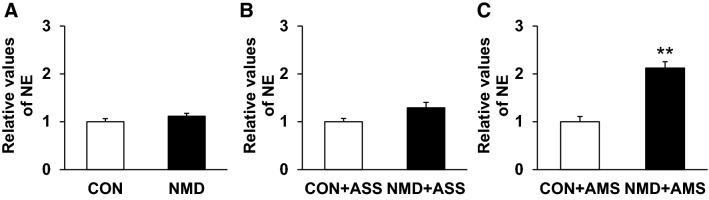
Multiple stressors increased norepinephrine concentration in blood plasma of NMD rats at 6 weeks. **A** NMD did not significantly alter the NE concentration compared with controls. **B** NMD + ASS did not significantly alter the NE concentration compared with CON + ASS. **C** NMD + AMS markedly enhanced the NE concentration compared with CON + AMS. **P < 0.01, two-sample *t* test.

NE is one of the important molecules involved in the regulation of stress responses [18, 20, 32]. In the present experiments, we demonstrated that there was no significant difference in NE concentrations in the plasma between control and NMD groups (*P* > 0.05, two-sample *t* test, Fig. 3A). There was also no significant difference in NE concentration in the plasma between the CON + ASS and NMD + ASS groups (*P* > 0.05, two-sample *t* test, Fig. 3B). However, there was a significant difference in NE concentrations in the plasma between CON + AMS and NMD + AMS rats, indicating that AMS remarkably increased the NE levels (*P* < 0.01, two-sample *t* test, Fig. 3C).

### AMS Enhances Excitability of Colon DRG Neurons in NMD Rats

**Fig. 4:**
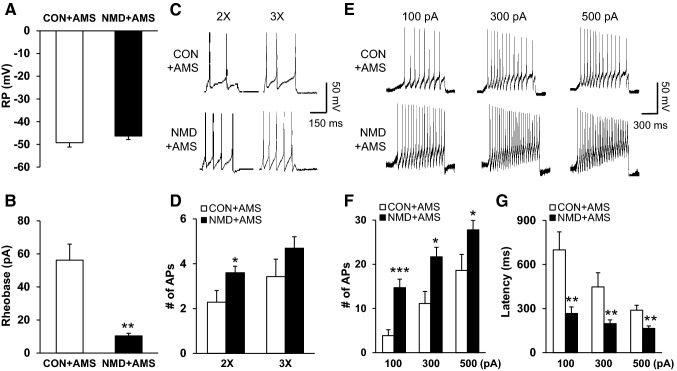
NMD and AMS enhanced colon DRG neuronal excitability. **A** NMD + AMS depolarized the resting membrane potential (RP) in DRG neurons. **B** NMD + AMS resulted in a marked reduction of the rheobase in DRG neurons. **C** Representative traces of action potentials (APs) induced by 300 ms depolarizing current injection at 2× rheobase (left) and 3× rheobase (right) in DRG neurons from CON + AMS and NMD + AMS rats under current-clamp. **D** Bar graph showing a significant increase in average numbers of APs elicited by 2× rheobase current injection in NMD + AMS rats compared with CON + AMS rats. **E** Representative traces of APs induced by 1000 ms depolarizing current injection at 100 pA, 300 pA, and 500 pA ramp stimulation in DRG neurons from CON + AMS and NMD + AMS rats under current-clamp. **F** Bar graph showing a significant increase in average numbers of APs evoked by 100 pA, 300 pA, and 500 pA ramp stimulation in NMD + AMS rats compared with CON + AMS rats. **G** Bar graph showing a significant decrease in latency of APs evoked by 100 pA, 300 pA, and 500 pA ramp stimulation in NMD + AMS rats compared with CON + AMS rats. NMD + AMS, *n* = 10 cells, *P < 0.05, **P < 0.01, ***P < 0.001 compared with CON + AMS, *n* = 8 cells, Mann-Whitney test and two-sample *t* test.

To determine the effects of NMD + AMS on the excitability of colon DRG neurons, whole-cell patch clamp recordings were carried out. The resting membrane potentials of T_13_-L_2_ DRG neurons were − 49.25 ± 1.92 mV (*n* = 8 cells) and − 46.3 ± 1.59 mV (*n* = 10 cells) for CON + AMS and NMD + AMS rats, respectively (Fig. 4A). After statistical analysis, there was no significant difference in resting membrane potentials between CON + AMS and NMD + AMS rats (*P* > 0.05, two-sample *t* test). Rheobase and firing patterns in response to current stimulation were also recorded. The average rheobases of colon DRG neurons were 56 ± 9.6 pA (*n* = 8 cells) from CON + AMS rats and 13 ± 2.1 pA (*n* = 10 cells) from the corresponding NMD + AMS rats (Fig. 4B, *P* < 0.01, Mann-Whitney test). The number of action potentials (APs) in response to 2× rheobase current stimulation was significantly increased in DRG neurons from the NMD + AMS group (Fig. 4C and D, *P* < 0.05, two-sample *t* test). However there was no significant difference in the number of APs evoked by 3× rheobase current stimulation (Fig. 4C and D, *P* > 0.05, two-sample *t* test). In addition, we counted the numbers of APs induced by 100 pA, 300 pA and 500 pA ramp current stimulation (Fig. 4E and F). The numbers of APs in response to 100 pA, 300 pA, and 500 pA ramp current stimulation differed significantly between CON + AMS and NMD + AMS rats (*P* < 0.05, *P* < 0.001, two-sample *t* test). At the same time, the latency of APs evoked by 100 pA, 300 pA, and 500 pA ramp current stimulation was significantly lower in the NMD + AMS group than in the CON + AMS group (Fig. 4G, *P* < 0.01, Mann-Whitney test), indicating that the neuronal excitability was enhanced in NMD rats after AMS treatment.

### AMS Upregulates the Expression of β_2_ Adrenergic Receptors in DRGs

**Fig. 5:**
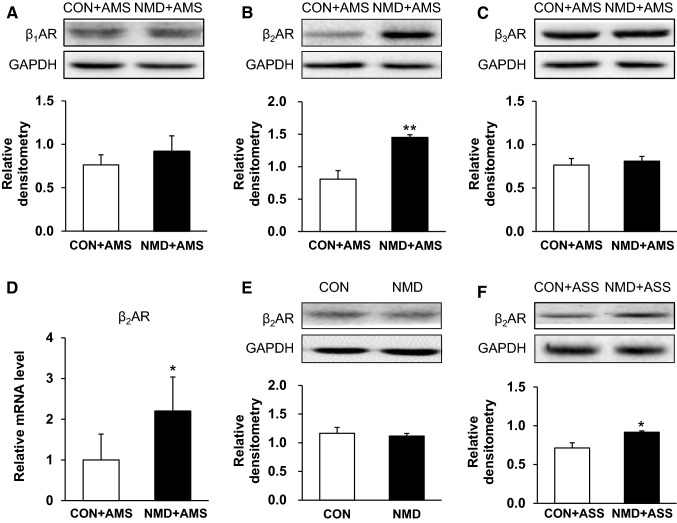
Multiple stressors upregulated expression of β_2_ adrenergic receptors (ARs) in DRGs of NMD rats at 6 weeks. **A** NMD + AMS did not change the expression of β_1_ ARs in DRGs between CON + AMS and NMD + AMS rats. **B** NMD + AMS significantly upregulated the expression of β_2_ receptors when compared with CON + AMS groups. **C** NMD + AMS did not change the expression of β_3_ receptors between CON + AMS and NMD + AMS rats. **D** NMD + AMS significantly enhanced the mRNA level of β_2_ receptors when compared with CON + AMS groups. **E** NMD did not alter the expression of β_2_ receptors when compared with CON groups at 6 weeks. **F** NMD + ASS significantly enhanced the expression of β_2_ receptors when compared with CON + ASS groups. *P < 0.05, **P < 0.01, two-sample *t* test.

Then we assessed the protein expression of adrenergic receptors beta 1, 2, 3 (β_1, 2, 3_) after AMS (Fig. 5A–C). The relative levels of β_2_ receptor proteins were 1.17 ± 0.10 (*n* = 3 rats) and 1.11 ± 0.14 (*n* = 4 rats) from CON rats and NMD rats, respectively (Fig. 5E), with no significant difference between the two groups (*P* > 0.05, two-sample *t* test), which is consistent with the above behavioral studies. The relative values of β_2_ receptor proteins were 0.81 ± 0.13 (*n* = 4 rats) and 1.45 ± 0.04 (*n* = 3 rats) in the CON + AMS and NMD + AMS rats, respectively (Fig. 5B, *P* < 0.01, two-sample *t* test). However, there was no significant difference in β_1_ and β_3_ receptor protein levels between CON + AMS and NMD + AMS rats (Fig. 5A and C, *P* > 0.05, two-sample *t* test). Furthermore, the relative mRNA levels of β_2_ receptor were 1.00 ± 0.63 (*n* = 5 rats) and 2.20 ± 0.83 (*n* = 6 rats) in CON + AMS and NMD + AMS rats, respectively (Fig. 5D, *P < 0.05, two-sample *t* test). Besides, the relative values of β_2_ receptor proteins were 0.71 ± 0.07 (*n* = 4 rats) and 0.92 ± 0.02 (*n* = 4 rats) in CON + ASS and NMD + ASS rats, respectively (Fig. 5F) (*P < 0.05, two-sample *t* test).

### Treatment with β_2_ Receptor Antagonist Reverses Visceral Hypersensitivity

**Fig. 6:**
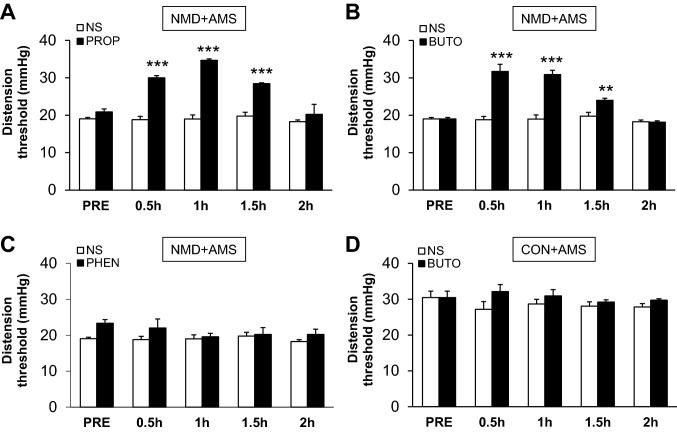
Norepinephrine β_2_ receptor antagonist reversed the visceral pain caused by NMD and AMS. **A** The NE β receptor antagonist propranolol (PROP, 3 mg/kg) significantly reduced the visceral hyperalgesia induced by NMD + AMS in a time-dependent manner. **B** The β_2_ receptor antagonist butoxamine (BUTO, 5 mg/kg) also reversed the visceral hyperalgesia induced by NMD + AMS in a time-dependent manner. **C** The α receptor antagonist phentolamine (PHEN, 3 mg/kg) had no effect on the visceral hyperalgesia induced by NMD + AMS. **D** BUTO (5 mg/kg) had no effect on CON + AMS rats. **P < 0.01, ***P < 0.001, Tukey *post-hoc* test following two-way repeated measures ANOVA. Note that the NS group in panels** A**–**C** used the same rats.

Next we determined whether AR antagonists can reverse chronic visceral hyperalgesia. Injection of PROP, a non-selective β receptor antagonist, significantly reversed visceral hyperalgesia in a time-dependent manner (Fig. 6A, NS: *n* = 12; PROP: *n* = 3, *P* < 0.001, compared with NS, Tukey *post-hoc* test following two-way repeated measures ANOVA). Further, injection of BUTO, a β_2_ receptor antagonist, significantly raised the DT of NMD + AMS rats (Fig. 6B, *n* = 12 rats/group, *P* < 0.05, *P* < 0.001, Tukey *post-hoc* test following two-way repeated measures ANOVA). However, injection of PHEN, an antagonist of α receptors, had no effect on the visceral hyperalgesia induced by AMS (Fig. 6C, NS: *n* = 12; PHEN: *n* = 3, *P* > 0.05, Tukey *post-hoc* test following two-way repeated measures ANOVA). In addition, there was no significant effect of BUTO at the same dose on the DT of CON + AMS rats (Fig. 6D, *n* = 5 rat/group, *P* > 0.05, Tukey *post-hoc* test following two-way repeated measures ANOVA). These data suggested that β_2_ receptors are involved in the development of the visceral hypersensitivity induced by AMS in NMD rats.

### Treatment with β_2_ Receptor Antagonist Reverses Neuronal Hyperexcitability

**Fig. 7:**
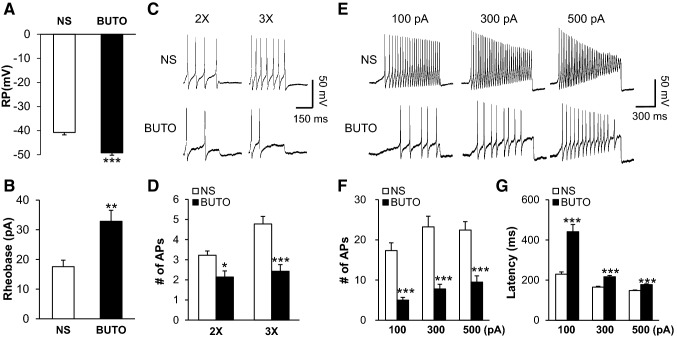
A β_2_ receptor antagonist reversed the enhanced excitability. **A** Administration of BUTO (5 mg/kg, once daily for 7 consecutive days) markedly reduced the resting potential (RP) increase induced by NMD + AMS. **B** BUTO significantly increased the rheobase decrease induced by NMD + AMS. **C** Representative traces of action potentials (APs) induced by 300 ms depolarizing current injection at 2× (left) and 3× (right) rheobase in DRG neurons from NS- and BUTO-treated NMD + AMS rats under current-clamp. **D** Bar graph showing a significant decrease in numbers of APs induced by 2× and 3× rheobase in BUTO-treated NMD + AMS rats, compared with NS-treated NMD + AMS rats. **E** Representative traces of APs induced by 1000 ms depolarizing current injection as 100 pA, 300 pA, and 500 pA ramps in DRG neurons from NS- and BUTO-treated NMD + AMS rats under current-clamp. **F** Bar graph showing a significant decrease in average numbers of APs evoked by 100 pA, 300 pA, and 500 pA ramps in BUTO-treated NMD + AMS rats, compared with NS-treated NMD + AMS rats. **G** Bar graph showing a significant increase in AP latency evoked by 100 pA, 300 pA, and 500 pA ramps in BUTO-treated NMD + AMS rats, compared with NS-treated NMD + AMS rats. BUTO, *n* = 13 cells, *P < 0.05, **P < 0.01, ***P < 0.001 compared with NS, *n* = 9 cells, Mann-Whitney test and two-sample *t* test.

To investigate the role of β_2_ receptors in NMD + AMS rats, we injected the β_2_ receptor antagonist BUTO once daily for 7 consecutive days to assess its influence on the excitability of colonic T_13_–L_2_ DRG neurons. The resting membrane potentials were − 40.78 ± 0.38 mV (*n* = 9 cells) in NS rats and − 49.23 ± 1.11 mV (*n* = 13 cells) in BUTO rats (Fig. 7A, *P* < 0.001, Mann-Whitney test). The average rheobases were 18 ± 2 pA (*n* = 9 cells) in NS rats and 33 ± 4 pA (*n* = 13 cells) in BUTO rats (Fig. 7B, *P* < 0.01, Mann-Whitney test). The numbers of APs in response to 2× and 3× rheobase current stimulation were significantly lower in DRG neurons after BUTO treatment (Fig. 7C, D, *P* < 0.05, *P* < 0.001, Mann-Whitney test and two-sample *t* test). In addition, we counted the number and measured the latency of APs induced by 100 pA, 300 pA, and 500 pA ramp current stimulation (Fig. 7E–G). The numbers of APs were significantly decreased and their latency was markedly increased (*P* < 0.001, Mann-Whitney test and two-sample *t* test). These results demonstrated that BUTO decreased the neuronal excitability enhanced by NMD and AMS exposure.

## Discussion

In the present experiments, we demonstrated that NMD rats exhibited reduced thresholds and increased abdominal withdrawal reflex scores to CRD when compared with age-matched control rats after exposure to multiple stressors as adults. This indicates that a combination of NMD and AMS exacerbated the symptoms by enhancing visceral hypersensitivity in NMD rats at the age of 6 weeks. This also supports an idea that NMD puts such rats at risk when they grew to the age of 6 weeks although these rats do not show any visceral hypersensitivity as reported previously [31]. Although these rats are more sensitive to multiple stressors at the age of 6 weeks than age-matched control rats, this does not mean that these NMD rats are sensitive to any environmental stimulus, since the CRS alone did not induce any visceral response. Environmental stimuli have to reach a minimal threshold to induce visceral hypersensitivity. Although we did not define the minimal threshold for NMD rats, our data might have clinic relevance in that an adverse neonatal stimulus followed by adult stressors aggravates the symptoms of IBS or/and shortens the time window to induce visceral hypersensitivity.

The finding of enhanced visceral sensitivity was strongly supported by the enhanced neuronal excitability in NMD rats followed by AMS. By whole-cell patch clamp recording, we showed that the excitability of DRG neurons was remarkably enhanced in NMD rats followed by AMS when compared with that of age-matched control rats followed by AMS. Our electrophysiological data provide a cellular mechanism underlying the enhanced visceral pain behaviors. Of note is that the ionic basis for this enhanced cellular excitability needs further investigation.

In the present experiments, we focused on the mechanism by which AMS produced visceral hypersensitivity and neuronal hyperexcitability in NMD rats at the age of 6 weeks. A recent study has shown that chronic stress involves NE release, AR expression, and/or the activation of intermediates in AR-induced signaling, thus contributing to the pathology of many immune-mediated diseases [33]. Adrenergic activation is involved in neuropathic and inflammatory pain states [34, 35]. Our previous studies showed that neonatal colonic inflammation or heterotypic intermittent stress increases the NE concentration in blood plasma without alterations in β_2_ AR expression in DRGs [14, 20]. A new finding in the present study was that AMS not only enhanced the plasma NE levels but also increased the expression of β_2_ ARs in DRGs at 6 weeks. This discrepancy might be due to the different stimulus protocols used. The neonatal colonic inflammation model was established only by one colonic stimulus in the neonate. The heterotypic intermittent stress model was induced by stressors only in adulthood [14, 17]. However, the present model was established by a combined stress protocol in both neonates and adults. This combination of stimuli might be more relevant to the clinic situation. Therefore, our study also provides a good animal model to better mimic the clinic situation in patients with IBS, thus providing a better basis on which to investigate the mechanisms of visceral pain.

We showed that β_2_ ARs played an important role in the peripheral nervous system. The expression of β_2_ ARs at the protein and mRNA levels was remarkably upregulated in NMD rats with AMS while the expression of β_1_ and β_3_ ARs was not altered greatly. Inhibition of β_2_ ARs by PROP or BUTO enhanced the CRD threshold in a time-dependent manner while inhibition of α adrenergic receptors by PHEN did not affect the CRD threshold. Furthermore, application of BUTO also reversed the enhanced hypersensitivity of DRG neurons. This anti-nociceptive effect was specific since BUTO did not have any effect on age-matched control rats with AMS. These data demonstrated that enhanced NE and β_2_ adrenergic signaling plays an important role in increasing the visceral hypersensitivity in rats with NMD and AMS. Of note is that the protein expression of β_2_ ARs in NMD rats followed by ASS was also significantly increased. However, the NE level in the plasma was not altered remarkably. This may explain why the NMD rats followed by ASS did not show enhanced visceral sensitivity. In addition, we do not know whether AMS can exacerbate the visceral hypersensitivity of NMD rats at the age of 7 weeks, although NMD rats at this age already exhibit enhanced visceral hypersensitivity. In the present study, we only focused on the peripheral mechanisms; the central mechanisms, such as spinal synaptic plasticity [36], deserve further investigation.

In summary, we demonstrated that NMD followed by AMS increased visceral hypersensitivity in association with an elevation of the NE concentration in plasma, the expression of β_2_ ARs in DRGs, and the neuronal excitability of colonic DRG neurons. Blockade of β_2_ ARs attenuated visceral hypersensitivity to colorectal distension and neuronal hyperexcitability. Together with our previous studies, our data provide additional evidence to support the idea that the NE–β_2_ signaling pathway plays an important role in the development of visceral hypersensitivity. This study might shed light on the pathogenesis of visceral hypersensitivity imposed by environmental stress during early and adult life. AR inhibitors might serve as alternates to relieve abdominal pain in patients with IBS.
